# Clinical Presentation and Treatment of a Patient With Prurigo Pigmentosa

**DOI:** 10.7759/cureus.94225

**Published:** 2025-10-09

**Authors:** Dana Rzek, Imge Hulur

**Affiliations:** 1 Dermatology, Hudson Dermatology, Kingston, USA

**Keywords:** ketogenesis, ketogenic diet, keto rash, ketosis, nagashima disease, prurigo pigmentosa

## Abstract

Prurigo pigmentosa is a rare inflammatory condition whose exact etiology is unknown; however, it is often misdiagnosed as eczema, contact dermatitis, or confluent and reticulated papillomatosis. A healthy 18-year-old male presented to the clinic with prurigo pigmentosa that developed after starting a ketogenic diet and intermittent fasting. The diagnosis was based on clinical and histopathologic findings. Treatment with oral minocycline and discontinuation of the diet led to complete resolution with no subsequent recurrence.

## Introduction

Prurigo pigmentosa (PP) is an inflammatory dermatosis characterized by pruritic erythematous papules that coalesce to form a reticulated pattern predominantly on the back, chest, and neck that resolves with hyperpigmentation. We present a ketogenic diet-associated case of PP in an 18-year-old male of South Asian descent. This case report aimed to raise awareness of this condition that is often misdiagnosed due to its rarity, resemblance to other dermatoses, and variable histopathologic findings.

This condition was first described by Masaji Nagashima in 1971. At that time, prurigo pigmentosa was typified as erythematous and papulovesicular lesions that are pruritic and often merge into a reticular configuration. The histopathology is variable and dependent on the stage of the lesions. Early lesions begin as a superficial and perivascular infiltrate of neutrophils that diffuse into spongiotic vesicles. The lesions later develop into necrotic keratinocytes, and as resolution occurs, hyperpigmentation is left behind in a distinctive netlike pattern. Based on clinical and histopathological findings, it is hypothesized that the pathogenesis of this dermatosis is associated with ketosis.

## Case presentation

**Figure 1:**
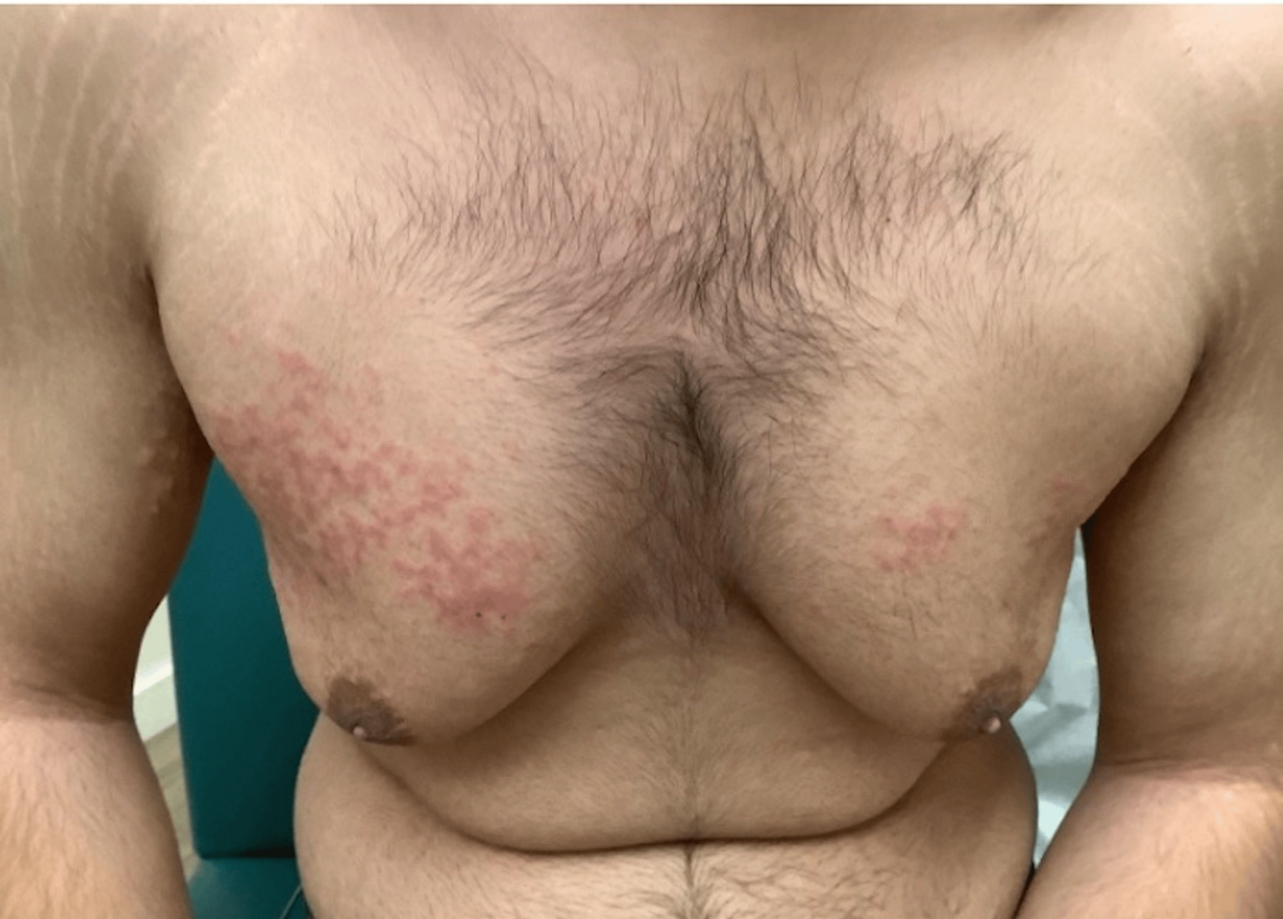
Erythematous papules and plaques in a reticulated pattern.

An 18-year-old male presented to our clinic with a six-month history of an intensely pruritic rash localized to his chest. The patient denied any systemic symptoms and was not using any new medications or personal care products. The appearance of the rash coincided with the adoption of a strict ketogenic diet; during that time, he stopped consuming carbohydrates and practiced intermittent fasting, and had lost approximately 22% of his body weight. Gynecomastia was partly manifested due to concomitant weight loss. The patient was initially seen in our clinic three months later and prescribed triamcinolone 0.1% acetonide cream for possible contact dermatitis. This provided temporary relief of his pruritus but did not improve the appearance of the rash. He was otherwise healthy with no history of diabetes and no family history of comparable cutaneous conditions. Physical examination revealed reticulated erythematous papules coalescing into plaques localized to the chest (Figure 1).

**Figure 2:**
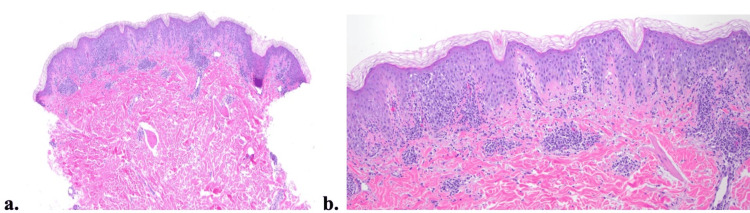
Histopathology of prurigo pigmentosa. Punch biopsy revealed interface dermatitis with a superficial dermal perivascular lymphocytic infiltrate. H&E (a) 4x and (b) 10x.

A 4 mm punch biopsy for microscopic examination was obtained from the right medial inferior chest (Figures 2a, 2b). Hematoxylin-eosin staining revealed interface dermatitis with a superficial dermal perivascular lymphocytic infiltrate; the presence of neutrophils and necrotic keratinocytes had not been documented.

Based on the clinical history, appearance, and supportive histologic findings, a diagnosis of prurigo pigmentosa (PP) was made. The patient was advised to avoid a ketogenic diet and was successfully treated with minocycline 100 mg twice daily for four weeks, with resolution of lesions. The recurrence-free period was limited to only four months; to confirm sustained remission, a longer follow-up would be needed.

## Discussion

PP is a rare inflammatory condition whose exact etiology is unknown, despite being often associated with ketogenic diets and ketosis, and is commonly misdiagnosed. Dr. Masaji Nagashima first documented it in 1971 [1,2]. This dermatosis has been referenced in literature as “Nagashima disease” or “keto rash” [2]. It was believed that this condition was endemic to Eastern Asia; however, as low-carb/ketogenic diets became more popular, the condition became more recognizable across the globe [3]. Since more recent studies show that PP can be found in varying ethnicities, it can be inferred that this condition is underdiagnosed because of minimal awareness [3].

Bile, which is produced by the liver, contains salts that act as emulsifiers when dietary fats are consumed, facilitating better absorption into the bloodstream [4,5]. If there is not a sufficient amount of bile or enzymes, malabsorption, vitamin deficiencies, gut microbiome imbalances, and inflammation may occur [6]. The patient’s PP condition is likely associated with ketosis. Considering glucose is limited during ketogenesis, the liver produces ketone bodies to serve as the body’s alternative energy source [7]. With strict ketogenic diets, there is a sudden elevation of ketones in the body, which briefly disrupts cellular homeostasis and likely increases oxidative stress within keratinocytes [8,9]. When inflammation occurs, there is a significant increase in matrix metalloproteinases (MMPs), which are involved in cleaving components of the extracellular matrix. In doing so, MMPs release the stored cytokines and growth factors, recruiting and guiding leukocytes to the site of inflammation, amplifying the response [10-12]. Minocycline is lipophilic, and due to its unique chemical structure, it can inhibit the activation of MMPs and other enzymes, thereby dampening inflammation [13-16].

PP is characterized by an initial inflammatory stage with intensely pruritic erythematous papules in a reticulate distribution, which later resolves into a hyperpigmented stage [2]. Histopathologic findings of PP depend on the stage of the lesion. Early lesions exhibit spongiosis and papillary dermal edema, accompanied by perivascular neutrophils and scattered necrotic keratinocytes [2,17,18]. Over time, lymphocytes become increasingly prominent, and within a few lesions, eosinophils have been noted to predominate over the neutrophils present in a dermal infiltrate that assumes a lichenoid pattern; later, melanophages predominate [2,17,18]. Lesions can be mistaken for eczema and contact dermatitis. However, these conditions respond to topical corticosteroids. PP may also be confused with confluent and reticulated papillomatosis (CARP) due to its appearance on the back, neck, and trunk, as well as its response to oral minocycline [17]. However, CARP is usually asymptomatic and lacks the inflammatory erythematous papules seen in PP [19].

## Conclusions

PP is a rare condition whose etiology is not yet fully understood. While PP was previously thought to be endemic to Eastern Asia, recent case reports indicate that PP can affect individuals from diverse ethnic backgrounds. The purpose of this case report was to raise awareness of a dermatological condition that, for this patient, was triggered by dietary restrictions. This case report aimed to highlight the effectiveness of treating the condition with oral minocycline and discontinuation of the ketogenic diet. Based on limited case reporting and awareness, it is evident that PP needs further investigation for management and prevention, especially as low-carb/ketogenic diets become more popular.

## References

[1] Nagashima M (1978). Prurigo pigmentosa: clinical observations of our 14 cases. J Dermatol.

[2] Shaker N, Sathe NC (2025). Prurigo pigmentosa. StatPearls [Internet].

[3] Mufti A, Mirali S, Abduelmula A, McDonald KA, Alabdulrazzaq S, Sachdeva M, Yeung J (2021). Clinical manifestations and treatment outcomes in prurigo pigmentosa (Nagashima disease): a systematic review of the literature. JAAD Int.

[4] Dave HD, Shumway KR, Al Obaidi NM (2025). Physiology, biliary. StatPearls [Internet].

[5] Hundt M, Basit H, John S (2025). Physiology, bile secretion. StatPearls [Internet].

[6] Omer E, Chiodi C (2024). Fat digestion and absorption: normal physiology and pathophysiology of malabsorption, including diagnostic testing. Nutr Clin Pract.

[7] Hwang CY, Choe W, Yoon KS, Ha J, Kim SS, Yeo EJ, Kang I (2022). Molecular mechanisms for ketone body metabolism, signaling functions, and therapeutic potential in cancer. Nutrients.

[8] Laffel L (1999). Ketone bodies: a review of physiology, pathophysiology and application of monitoring to diabetes. Diabetes Metab Res Rev.

[9] Youm YH, Nguyen KY, Grant RW (2015). The ketone metabolite β-hydroxybutyrate blocks NLRP3 inflammasome-mediated inflammatory disease. Nat Med.

[10] Kanikarla-Marie P, Jain SK (2016). Hyperketonemia and ketosis increase the risk of complications in type 1 diabetes. Free Radic Biol Med.

[11] Nissinen L, Kähäri VM (2014). Matrix metalloproteinases in inflammation. Biochim Biophys Acta.

[12] Ramesh G, MacLean AG, Philipp MT (2013). Cytokines and chemokines at the crossroads of neuroinflammation, neurodegeneration, and neuropathic pain. Mediators Inflamm.

[13] Asadi A, Abdi M, Kouhsari E (2020). Minocycline, focus on mechanisms of resistance, antibacterial activity, and clinical effectiveness: back to the future. J Glob Antimicrob Resist.

[14] Garrido-Mesa N, Zarzuelo A, Gálvez J (2013). Minocycline: far beyond an antibiotic. Br J Pharmacol.

[15] Griffin MO, Fricovsky E, Ceballos G, Villarreal F (2010). Tetracyclines: a pleitropic family of compounds with promising therapeutic properties. Review of the literature. Am J Physiol Cell Physiol.

[16] Nikodemova M, Duncan ID, Watters JJ (2006). Minocycline exerts inhibitory effects on multiple mitogen-activated protein kinases and IκBα degradation in a stimulus-specific manner in microglia. J Neurochem.

[17] Beutler BD, Cohen PR, Lee RA (2015). Prurigo pigmentosa: literature review. Am J Clin Dermatol.

[18] Böer A, Misago N, Wolter M, Kiryu H, Wang XD, Ackerman AB (2003). Prurigo pigmentosa: a distinctive inflammatory disease of the skin. Am J Dermatopathol.

[19] Shevchenko A, Valdes-Rodriguez R, Hsu S, Motaparthi K (2018). Prurigo pigmentosa: case series and differentiation from confluent and reticulated papillomatosis. JAAD Case Rep.

